# Delineating family needs in the transition from hospital to home for children with medical complexity: part 1, a meta-aggregation of qualitative studies

**DOI:** 10.1186/s13023-023-02942-9

**Published:** 2023-12-12

**Authors:** L. van de Riet, M. W. Alsem, E. C. van der Leest, F. S. van Etten-Jamaludin, J. M. Maaskant, J. B. M. van Woensel, C. D. van Karnebeek

**Affiliations:** 1 Department of Pediatric Intensive Care, Amsterdam UMC, University of Amsterdam, Meibergdreef 9, Amsterdam, The Netherlands; 2 Amsterdam Reproduction and Development Research Institute, Amsterdam, The Netherlands; 3 On Behalf of the Transitional Care Unit Consortium, Amsterdam, The Netherlands; 4 Department of Rehabilitation, Amsterdam Movement Sciences, Amsterdam UMC, University of Amsterdam, Meibergdreef 9, Amsterdam, The Netherlands; 5 Medical Library AMC, Amsterdam UMC, University of Amsterdam, Meibergdreef 9, Amsterdam, The Netherlands; 6 Department of Pediatrics, Amsterdam UMC, University of Amsterdam, Meibergdreef 9, Amsterdam, The Netherlands; 7 Emma Center for Personalized Medicine, Departments of Pediatrics and Human Genetics, Amsterdam Gastro-Enterology Endocrinology and Metabolism, Amsterdam UMC, University of Amsterdam, Meibergdreef 9, Amsterdam, The Netherlands

**Keywords:** Children with medical complexity, Hospital-to-home transition, Care pathway, Parental needs, Qualitative research, Systematic review, Meta-aggregation

## Abstract

**Background:**

Advances in diagnostic and therapeutic interventions for rare diseases result in greater survival rates, with on the flipside an expanding group of children with medical complexity (CMC). When CMC leave the protective hospital environment to be cared for at home, their parents face many challenges as they take on a new role, that of caregiver rather than care-recipient. However, an overview of needs and experiences of parents of CMC during transition from hospital-to-home (H2H) is lacking, which hampers the creation of a tailored H2H care pathway. Here we address this unmet medical need by performing a literature review to systematically identify, assess and synthesize all existing qualitative evidence on H2H transition needs of CMC parents.

**Methods:**

An extensive search in Medline, PsychINFO and CINAHL (up to September 2022); selection was performed to include all qualitative studies describing parental needs and experiences during H2H transition of CMC. All papers were assessed by two independent investigators for methodological quality before data (study findings) were extracted and pooled. A meta-aggregation method categorized the study findings into categories and formulated overarching synthesized findings, which were assigned a level of confidence, following the ConQual approach.

**Results:**

The search yielded 1880 papers of which 25 met eligible criteria. A total of 402 study findings were extracted from the included studies and subsequently aggregated into 50 categories and 9 synthesized findings: (1) parental empowerment: shifting from care recipient to caregiver (2) coordination of care (3) communication and information (4) training skills (5) preparation for discharge (6) access to resources and support system (7) emotional experiences: fatigue, fear, isolation and guilt (8) parent-professional relationship (9) changing perspective: finding new routines and practices. The overall ConQual Score was low for 7 synthesized findings and very low for 2 synthesized findings.

**Conclusions:**

Despite the variability in CMC symptoms and underlying (rare disease) diagnoses, overarching themes in parental needs during H2H transition emerged. We will augment this new knowledge with an interview study in the Dutch setting to ultimately translate into an evidence-based tailored care pathway for implementation by our interdisciplinary team in the newly established *‘Jeroen Pit Huis’*, an innovative care unit which aims for a safe and sustainable H2H transition for CMC and their families.

**Supplementary Information:**

The online version contains supplementary material available at 10.1186/s13023-023-02942-9.

## Introduction

The life expectancy of children with incurable conditions has improved significantly over the past decades, which resulted in a substantial increase in both the number of children with chronic conditions and their average lifespan [1–4]. Within healthcare institutions, this has led to a shift in focus from *cure* to *care*. Some of these chronically ill children are referred to as children with medical complexity (CMC), defined by Cohen et al. as children with one or more complex chronic illness requiring specialized care, functional disabilities, high healthcare utilization, and high family-identified needs [5]. The CMC population is relatively small, but since many of them rely on technology and 24/7 nursing care, their use of healthcare resources is substantial [2, 6, 7].

Once CMC are medically stable, the goal of care for both their parents and professionals is to reunite them with their community [8, 9]. However, the transition between the protective hospital environment and home, where parents suddenly take on a new caregiving role, is major and creates a gap that is difficult to bridge. It requires constant adaptation to new situations and forces parents and professionals to alternate their individual roles and responsibilities [10, 11]. The heterogeneity of CMC and their family situation inevitably leads to care needs that are constantly changing, which makes them difficult to address with a *one size fits all* care program [10, 12, 13]. Moreover, their frail condition predisposes CMC to complications leading to unplanned and often lengthy hospital admissions [2, 4], that compromise the well-being of both child and family [14–18].

Ways to improve the transition of care between the hospital and home (H2H) for CMC families, has been investigated in recent years [8, 19–23]. For example, Hamline et al. stated in their meta-analysis that a better coordination of H2H care could potentially reduce readmissions and emergency room visits [24]. In addition, access to clear and accessible information would increase parental self-efficacy and thus enable a more sustainable H2H transition [25]. Yet, many interventions only seem to focus on a specific part of the H2H transition process and do not take sufficient account of its integral character. In order to develop a more sustainable H2H care pathway that covers all potential needs of CMC families, a proper understanding and delineation of these needs is crucial. However, an overview of these needs is currently lacking.

Therefore, the aim of this study is to provide a full overview of the existing evidence regarding the needs and experiences of parents of CMC during H2H transitions. By summarizing cross-study generalisations, the results of this meta-aggregation of qualitative studies will enable the development of a H2H care pathway, tailored to the needs of CMC families. This will contribute to a more sustainable H2H transition that will benefit the children, their families, and the professionals.

## Methods

We systematically identified and synthesized all primary qualitative research exploring the needs and experiences of CMC parents during H2H transitions, according to the Johanna Briggs Institute guidelines for the meta-aggregative approach [26]. A meta-aggregation is an integrative method of qualitative synthesis designed to summarise results of qualitative research while retaining the original wording and interpretation of the outcome. Summarizing common findings produces cross-study generalizations that could lead to recommendations for action [27, 28]. Our study protocol was published on PROSPERO [record number CRD42020196348].

### Search strategy and inclusion criteria

An extensive literature search was conducted in Medline, PsychINFO and CINAHL from start up to September 2022. See appendix 1 for full search strategy. To be considered eligible for inclusion, studies had to 1) be peer-reviewed, 2) be available in English or Dutch, 3) follow qualitative study designs such as, but not limited to phenomenology, ethnography or grounded theory (the qualitative components of mixed methods studies were also considered potentially eligible), and 4) describe H2H transition needs and experiences of parents of children aged 0–18 year with medical complexity as defined by Cohen et al. [5]. Studies that focused on the H2H transition of healthy children, the transition of care from paediatric care towards adult care, or solely on adult healthcare, were excluded from the review. In addition, studies that focused on transition towards a setting other than the home and studies that described the professional perspective of H2H care of CMC, were excluded as well.

### Selection of papers

Two reviewers (LR/MA or LR/CK) assessed all papers independently on eligibility for inclusion by primary screening of title and abstract, and if applicable, followed by full text evaluation (LR, EL). Disagreement was resolved through discussion or involvement of a third reviewer (MA, CK).

### Data extraction

Data extraction from the included papers was performed independently by two reviewers (LR, EL). Conflicting insights were resolved through discussion. If consensus could not be achieved, a third reviewer was involved (MA). The process of data extraction involved the following steps: Line-by-line coding was performed to extract the results from the included studies. The results or ‘themes’ from the original studies were labelled as *finding*s. If available, these *findings* were accompanied by a citation that informed the *finding.*

### Data synthesis and the meta-aggregation

Four reviewers (LR, EL, MA, CK) performed the meta-aggregation. First, *findings* were independently assembled by two reviewers (LR, EL) and subsequently categorized based on similarity in meaning. According to the Joanna Briggs Institute guidelines [26], a category had to consist of at least 2 *findings.* A third reviewer was involved (MA) to reach consensus at this stage. The final aggregative step was the definition of the overarching themes, the so-called *synthesized findings,* from the categories. This iterative process was performed jointly by the four reviewers, who reviewed the data extensively until they agreed on the final *synthesized findings*.

### Assessment of methodological quality and the ConQual approach

All included studies were critically appraised with the use of the Critical Appraisal Checklist for Qualitative Research from the Joanna Briggs Institute [29]. Two reviewers (LR/MA, LR/EL or LR/CK) performed this assessment independently and compared their results once the initial appraisal was completed by both parties. Discussion regarding differences followed until consensus was reached.

To help interpret the quality of the overall results, a ConQual score was assigned to each *synthesized finding* [26, 30]. This is a score ranging from high–moderate–low–very low, depending on a level of dependability and credibility. First, a dependability score was assigned to each of the included papers. The dependability score ranges from 0 to 5 and is based on five items from the Critical Appraisal Checklist for Qualitative Research, which address the appropriateness of conducting the study in terms of research aims and purpose [30]. Second, during data extraction, a level of credibility was assigned to each individual *finding* through a credibility score, which is based on the transparency and congruency of the results and rates a *finding* either as unequivocal (U), credible (C) or not supported (NS) [26, 30]. For example, in case of a clear statement that is illustrated by a citation from the original study, a *finding* was rated as unequivocal (U). If this was not the case, but a statement was otherwise elaborately explained, it was rated as credible (C). When U nor C apply and *findings* are not supported by the data, they could be rated as not supported (NS). Eventually, during the final steps of the meta-aggregation, these scores are combined to interpret the final results, which is shown in the ConQual Summary of Findings Table 1.

**Table 1:** ConQual summary of findings

Synthesized finding	Type of research	Dependability	Credibility	ConQual score^#^
1. Parental empowerment: shifting from care recipient to caregiver	Qualitative studies	Downgrade 1 level*	Downgrade 1 level^¥^	Low
2. Coordination of care	Qualitative studies	Downgrade 1 level*	Downgrade 1 level^¥^	Low
3. Communication and information	Qualitative studies	Downgrade 1 level*	Downgrade 1 level^¥^	Low
4. Training skills	Qualitative studies	Downgrade 1 level*	Downgrade 1 level^¥^	Low
5. Preparation for discharge	Qualitative studies	Downgrade 1 level*	Downgrade 1 level^¥^	Low
6. Access to resources and support system	Qualitative studies	Downgrade 1 level*	Downgrade 1 level^¥^	Low
7. Emotional experiences: fatigue, fear, isolation, and guilt	Qualitative studies	Downgrade 2 levels**	Downgrade 1 level^¥^	Very low
8. Parent-professional relationship	Qualitative studies	Downgrade 1 level*	Downgrade 1 level^¥^	Low
9. Changing perspective: finding new routines and practices	Qualitative studies	Downgrade 2 levels**	Downgrade 1 level^¥^	Very low

^#^ConQual score: Score ranging from highmderate–low–very low, depending on a level of dependability and credibility

^*^Downgraded one level due to mixed dependability scores (high and moderate) of primary studies. Common dependability issues across the included studies were for example no statement locating the researcher and/or no acknowledgement of their influence on the research

^**^Downgraded two levels due to mixed dependability scores (high, moderate, and low) of primary studies

^¥^Downgraded one level due to a mix of unequivocal and credible findings

### Software

Rayyan (a web and mobile app for systematic reviews, 2016) [31] was used to perform title-abstract screening and for finalizing the list of included studies. MaxQDA 2022 (VERBI software, 2021) was used to extract and synthesize data [32].

## Results

### Search and study selection

**Fig. 1:**
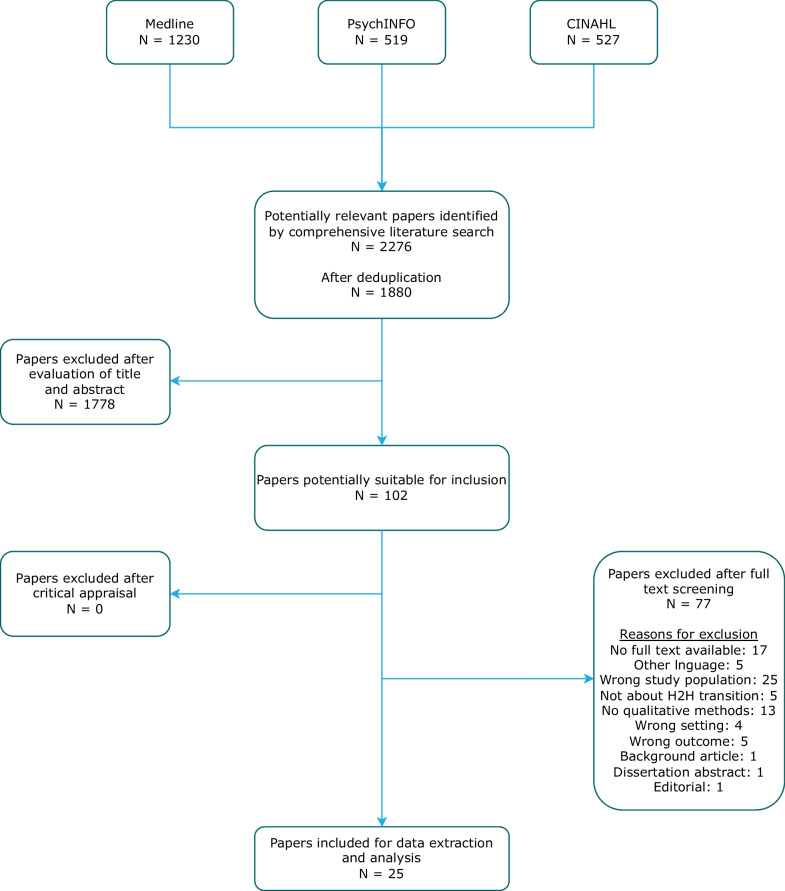
Flow diagram of search strategy

We identified 1880 potentially eligible papers of which 1778 were excluded based on screening of titles and abstracts. After full-text screening 102 additional papers were excluded, resulting in 25 papers [10, 22, 23, 33–54] that were included (Fig. 1, Additional file 2: Table S1). They represent a wide range of qualitative methodologies such as phenomenology, ethnography, participatory action research and Grounded Theory, which were conducted in various regions around the world (Northern Europa, Southern Europa, New Zealand, South America and several locations within the United States of America and Canada).

### Data extraction and synthesis

A total of 402 (partly overlapping) *findings* were extracted from these 25 papers, that all described the perspective of CMC parents in the H2H transition. The process of meta-aggregation involved the evaluation of similarity in meaning of these *findings* (n = 402) across the included studies, in order to synthesize them into categories (n = 50). These categories (n = 50) were subsequently aggregated into nine *synthesized findings.* Additional file 3: table S2 provides an overview of the results and includes a selection of the 402 *findings,* their level of credibility and illustrative citations. The nine *synthesized findings* are explained below. Additional file 4: table S3 illustrates the distribution of the nine *synthesized findings* across the 25 included papers.

### Assessment of methodological quality and the ConQual approach

Of the 402 *findings*, 207 were rated as unequivocal (U) and 195 were rated as credible (C). There were no not supported (NS) *findings*. As illustrated in the ConQual Summary of Findings, the overall ConQual Score was low for 7 synthesized findings and very low for 2 synthesized findings.

#### Synthesized findings

1. Parental empowerment: shifting from care recipient to caregiver

Providing care at home comes with a great deal of responsibility. Taking ownership and achieving a level of empowerment was described as a gradual process. Parents must **develop their own practical skills as well as go through an emotional transition** to gradually accept the responsibility that comes with their new role as caregiver. Building self-confidence and trusting their own instincts were considered important and developed over time. When this increase in confidence was well balanced, parents reported feeling they knew when to seek help as part of proactive care delivery. Mastering certain care tasks was considered a source of pride. **The learning process**, although despairing at times, **increased self-esteem** and should be encouraged by professionals as soon as possible. By becoming expert caregivers, some parents also wanted to advocate for other families facing the same problems. In addition, they wanted to help professionals improve the overall care delivery. They felt empowered.

2. Coordination of care

Coordination of care was mentioned to be important **throughout the entire transition process**, both during admission and at home. Parents appreciated to be actively involved in the planning and coordination of care. The presence of a comprehensive care plan was perceived as a useful tool to support continuity of care, and to communicate well with all the involved health professionals and institutions once home. Parents also **valued active follow up** by professionals, especially in the first days at home, for example a home visit or a telephone call. The lack of a clear point of contact for questions and the lack of cooperation between the healthcare organizations involved, were mentioned as obstacles in the coordination process. When asked about novel ideas to improve coordination of care, the support of a designated professional to help to coordinate appointments after discharge was considered helpful by parents.

3. Communication and information

Communication and information were often mentioned by parents as an important aspect of transitional care. They emphasized the importance of receiving **comprehensible** information in an understandable language avoiding medical jargon. Information inconsistent with the parents’ literacy level negatively impacted their education to become their child’s caregiver. Information had to be **reliable** as well. If not provided by professionals, parents explained that they would search information elsewhere, i.e. on the internet. During a hospital admission, it was common for parents to have to deal with many different healthcare professionals that could easily contribute to **inconsisten**t information about the care plan. Several parents indicated that they felt safe and in control when information about the child’s condition was **customised** and tailored to their specific family situation. Not knowing what to expect was challenging, but parents preferred honesty over false hope. This also included **clear expectations** about inevitable setbacks in the child’s clinical course. Some parents emphasized the need for the online availability of (medical) information upon discharge, in case they lost their discharge papers. The availability of other digital tools such as video chats, health portals, and **online educational videos** could be helpful tools to stimulate self-education.

4. Training skills

Training specific skills resulted in **a gradual learning curve** for parents, ideally starting early during the hospital stay and extending well after discharge. Parents emphasized the importance of **practicing** care tasks in a timely manner. They suggested scheduling protected time, separate from routine rounds and without the child present. Parents overcame (technical) difficulties over time and a gradual **education process tailored to the patient and family**, potentially resulted in safer and more competent care provision at home. **Anticipating the practicalities of the post-discharge situation** was an important part of the training process. Parents mentioned the positive effect of a dress rehearsal before discharge. Furthermore, they felt more confident if there were clear practical instructions about what measures they could take themselves if their child deteriorated at home. In addition, adequate anticipation on possible equipment related matters, such as professional electrical inspections and equipment installations, was considered important.

5. Preparation for discharge

According to parents, the moment of discharge was a big event in which several practical matters had to be considered. Not knowing how to organize things at home was a major stressor. The fear of the new situation was greatest for the care that took place at night. Practising this before discharge (rooming-in) was suggested to improve a sense of readiness. Arranging home care **equipment** was time consuming and equipment at home could be slightly different than the hospital equipment. Parents wished to envision their own homes with all the new equipment in it, to anticipate logistical issues before actual discharge. Some parents pointed out that arranging practicalities, such as proper **transportation** or the right type of funding, could delay the discharge process due to unforeseen costs and time. Additionally, coming home to medical bills added to the other stressors associated with transitioning home. **A timely inventory of financial resources** and the organisation of financial support if needed, may relieve this burden for parents.

6. Access to resources and support system

Arranging good quality home care took time and was difficult for parents. They often did not know how to **screen for qualified paid caregivers**. Moreover, a scarcity of them led to an increased level of stress, possible costs, and responsibility on the part of parents. **Proximity and access to good healthcare facilities** proved crucial in case of an emergency. **Availability of specific equipment and medication** could be a problem, especially in more rural areas where these are not always easily available. Several families reported having financial problems due to loss of work and unforeseen extra costs (frequent transportation, higher energy costs). In severe cases, financial difficulties forced families to prioritize child care over essential household items, such as a refrigerator, heating, and telephone services. Signing up for **financial aid programs** was a major challenge. In addition, the recruitment, training, and funding of professional caregivers at school was a source of contention between some families and paying agencies. Parents needed a support network to cope with CMC care. **Friends and family** were a huge help if they were educated and trained in the care. Another important form of support came from other parents in similar situations. **Peer support was a source of friendship, support, and helped parents to accept their new reality**. Occasionally parents found motivation and **meaning in religion**.

7. Emotional experiences: fatigue, fear, isolation, and guilt

Many CMC families experienced emotional difficulties associated with the H2H transition. Parents had to be prepared for some degree of **fatigue and possible feelings of disappointment** after discharge. Parents often felt **overwhelmed** and petrified in their first days at home, even though they experienced a sense of empowerment and readiness in the hospital. Reasons for parental **anxiety** ranged from neglecting other siblings to a fear of losing their child. Experienced parents compared caring for a CMC with being a first-time parent. Everything was new again, which reduced their self-confidence. A common response to fear was control. Parents were constantly alert and questioned decisions of (newly involved) professionals, especially when mistakes had been made. Parents often felt that others in their social network had little understanding of what they were going through, which could lead to fewer social interactions and **a sense of isolation**. Returning to work could improve social interactions, but at the same time reduce parental health and wellbeing due to sleep deprivation and distraction from the sick child’s needs at home. Some parents expressed **feelings of anger, guilt, and self-blame**, for example about the child being sick. They worried if they did something wrong. Other parents were angry about the disruption of the life as they planned it, but at the same time felt guilty about feeling angry about it. Finally, **the unknown future could be frightening** for parents. They expressed concern about their child’s perspective and their ability to cope with a disability. Parents found it difficult to think beyond the present moment and often compared their sick child with healthy peers. Believing their development was lagging, they feared that their child would be stigmatized or bullied in the future.

8. Parent-professional relationship

The relationship between parents and professionals plays an important role in the provision of care for CMC. **Equality** reinforced this special relationship. Parents expressed the importance of a personal, but also professional relationship with the involved professionals. Nurses were often the primary source of information and building a trusting relationship with them created a safe environment for parents to learn to care for their child. In addition to their own relationship with (home) professionals, parents indicated that **a trusting relationship between the sick child and a home nurse,** who noticed the individual needs of the child, was also very valuable. It made it easier for them to hand over some of the responsibility for the child to the professionals. **Continuity of care provided a welcome sense of familiarity.** A familiar face during follow-up appointments was highly appreciated by parents. In fact, it helped to distinguish between ‘normal’ symptoms or worsening of the disease and to determine if escalation of medical care was necessary. When they were more experienced, **parents wanted recognition** for their knowledge and acquired skills and to be part of the decision-making process. They sometimes felt that **they could take better care of their child than the home nurse**. Repeated discharge ‘tests’ and questioning about their child’s condition resulted in frustration, feelings of isolation and stress.

9. Changing perspective: finding (new) routines and practices

One of the biggest challenges for families during the H2H transition has been to change their perspective and find normalcy as a family (again). Parents needed to **adjust to having all the new equipment at home.** The equipment also made it extremely difficult for them to leave the house. Although many parents complemented their home nurses, their constant presence resulted in a **loss of privacy and control**, which was reported to be a source of stress by some families as well. Parents **drew strength from old routines and established new ones**, which was seen as an important preparation to go home and reduced parental stress. However, the **new routines could easily be disrupted**, for example by an unforeseen readmission. When parents were able to take their child home, they repeatedly mentioned the advantage of not having to constantly split up the family. Yet, once at home **parents have a dual role** by being both parent and caregiver. In addition, it proved to be a challenge to divide themselves between the sick child, other siblings, their partner, and a job. **The importance of self-care** to reduce parental anxiety and stress has been emphasized in several studies. Returning home increased the workload for many parents, resulting in chronic physical and mental fatigue. Some parents said they tried their best, but felt it was never good enough. **Adjusting to having a special need child**, while at the same time learning new skills, took time. Overall, when finding normalcy at home, many parents noticed a different sense of calm, better sleep, and less stress. They **enjoyed resuming normal daily activities with their family.**

## Discussion

In this study we present a comprehensive literature review of the needs and experiences of parents of CMC when they transition between the hospital and home. The meta-aggregation, in which we extracted and analysed the H2H transition needs and experiences from the 25 original studies, resulted in the formation of 9 overarching synthesized findings: 1) parental empowerment: shifting from care recipient to caregiver 2) coordination of care 3) communication and information 4) training skills 5) preparation for discharge 6) access to resources and support system 7) emotional experiences: fatigue, fear, isolation, and guilt 8) parent-professional relationship 9) changing perspective: finding new routines and practices. This knowledge is crucial to develop a care pathway, tailored to the needs of CMC families, that facilitates a sustainable H2H transition. Our results are similar to themes that emerged in a scoping review on the experiences of parents of preterm and acutely sick children during H2H transition; being involved in decision making, timely information about the transfer home, and support from family, friends or peers [55]. Both reviews show that parents also consider non-medical aspects of H2H transition care to be important, whereas more traditional care standards focus mainly on management of the disease [56]. This more holistic view on care delivery, expressed by parents, could potentially change the way we address CMC transitional care. The nine synthesized findings reflect two very important aspects of that care.

First, the variety of care needs of CMC families requires a form of healthcare that is flexible and adaptable to their unique and ever-changing family situations. However, the results of this meta-aggregation also imply that their H2H care needs are in fact not equally unique. Identifying and focusing on similarities between CMC families might ease H2H care provision for both parents and professionals. Encompassing the nine overarching synthesized findings into a care pathway, may provide a solid foundation for families to identify with, and for professionals to tailor their H2H interventions to.

Second, the results emphasize the need for a holistic approach, addressing both practical and emotional aspects of care, considering not only the needs of the CMC, but of the entire family. The role of the family has become important within pediatric healthcare, which increasingly consists of chronic care provision. Established models, such as family-centered care (FCC) and family-integrated care (FIC) place the patient and the family at the center of their own care delivery [57, 58] Indeed a large multicenter randomized trial by O’Brien et al. [57] examining the effects of FIC in neonatal intensive care units, showed positive outcomes for both child and family. FIC decreased parental stress and anxiety and had positive effects on the child’s overall health. However, in the context of H2H transitional care this requires not only the patient/parent, but also professionals to take on different roles, and demands collaboration with parents. Our results reinforce this idea, as many CMC parents want to be involved in the care and be acknowledged as their child’s expert. Still, this new-formed parents-professional partnership can only thrive when all parties involved feel confident.

An important concept in this shift to more holistic care is empowerment. Empowerment as a concept has been the topic of many research projects, which has not necessarily simplified its use, as Fumagalli et al. point out in their extensive literature review [59]. There happens to be a lot of ambiguity around the idea of empowerment and neighboring concepts such as engagement, activation, participation, and enablement. Empowerment can be interpreted as an emergent state in which patients play an active role in their own care. At the same time, it is often seen as the process leading toward this emergent state. Both definitions rely on the acquisition of two things: motivation (self-awareness, and attitude acquired through engagement) and ability (the skills and knowledge which one gains through enablement). In order to help both parents and professionals to truly feel empowered, it is important to know what is needed to achieve this. If enablement includes all components that lead to a better ability to cope with the ill child both in the hospital and at home, our results suggest that the combination of coordination of care, communication, peer support, and gradual learning are important factors. Simultaneously, the emotional process that CMC families go through influences their level of motivation and the way in which they feel engaged. By gaining more responsibility and knowledge, and adjusting to their new normal, parents will become (medical) experts of their own child. Still, this fluid concept is found to be disempowering in times in need of acute hospital care when the expertise of parents is often not acknowledged [60].

Improving H2H care is important, not only to provide the best possible support to CMC families, but also to optimally distribute the available resources in healthcare, in which there is currently an imbalance. Many CMC require complex and technology-assisted care for prolonged periods of time, which means that when they deteriorate at home, the only solution is to readmit them to a hospital, often a paediatric intensive care unit (PICU). For the last decades, PICUs that traditionally are designed to provide acute care, have been struggling with the distribution of their resources [2, 7]. A limited number of chronically ill children occupy acute beds for months, sometimes even years [6, 61]. Until families are able to provide this complex type of care at home themselves, they must rely on hospitals, nursing facilities and extensive home care. The process of empowerment that CMC families go through takes time. Time not spend at home as a family. A newly established care facility in the Netherlands, a Transitional Care Unit (TCU) called the *‘Jeroen Pit Huis’*, intends to mimic the home situation [62, 63]. In this TCU, CMC and their families can stay while practising in, and adapting to, their new reality until they are ready to transition home. A safety net of healthcare professionals helps parents to gradually take on their new role as their child’s primary caregiver.

### Strengths and limitations

We did not consider differences between individuals when aggregating our findings, which may be seen as a limitation of this study. Cultural, financial, and geographical variation might influence the perspective of parents and therefore lead to different perceptions of the care they received and the obstacles they faced during the H2H transition process. For example, one of the included studies in this meta-aggregation described different experiences between socially marginalized families and middle-class families [38]. The presence of a home nurse was perceived as a lack of privacy for the middle-class families, whereas the socially marginalized families experienced this contact as a decrease in social isolation. In addition, Lakshmanan et al. emphasized that only low-income families participated in their study and that their results may not be universal [43]. Another factor to consider when interpreting the different parental perceptions is language. Thirteen included papers specified which language was used in their studies, the other twelve papers did not. With the exception of one study that offered participants the option of using a second language [43], a good understanding of the country’s main language by the participants was a requirement in all other studies. This may reduce the representativeness of the study samples, as minorities were not included. While we did not take this inter-individual factor into account when including the studies, we believe that the scope of our search resulted in the representation of a great number of parents from different parts of the world. They represent a heterogeneous group of CMC families that offer a broad perspective on H2H transition. In addition, the nine overarching synthesized findings are based on cross-study similarities. Nevertheless, future research could focus more on specific subgroups and their experiences regarding transition care.

Another factor not taken into account, which may influence parental needs and experiences, was the duration and number of hospital admissions. It is known that the readmission rate under CMC is very high [6, 19, 61], and the transition process does not simply stop or start after discharge. Therefore, it would be difficult to define the exact duration. However, it may be an important factor, because a longer admission creates more time for training and preparation for discharge and our results show the importance of time to adjust to a new reality as well. Yet, a recent study by van der Perk et al. examining prognostic factors influencing parental empowerment after discharge, found that longer length of hospital stay did not exert a significant positive influence on parents [64]. Another limitation of the study is that it does not consider the influence of the etiological diagnosis of the child, the severity of the medical complexity, and the physical and mental functioning of the child and family.

### Conclusions and recommendations

While families of CMC are diverse and have different transitional care needs, this meta-aggregation of qualitative studies revealed overarching themes regarding parental needs in the H2H transition process. These results will enable the development of intervention programs that meet the specific H2H needs of CMC and their parents. We will augment this new knowledge with an interview study in the Dutch setting to ultimately translate into an evidence-based personalized H2H care pathway for implementation in our newly established TCU. The more CMC families are enabled and feel engaged, the better they will become empowered to eventually take their new role as primary caregiver, and feel confident while doing so.

### Supplementary Information

**Additional file 1:** PIC(o), inclusion criteria and search strategy.

**Additional file 2: Table S1.** Included study characteristics.

**Additional file 3: Table S2.** Overview of the synthesized findings, categories and a selection of study findings. Illustrated by citations.

**Additional file 4: Table S3.** Synthesized findings represented throughout the included papers.

## Data Availability

The dataset supporting the conclusions of this article is partly included in Additional file 2: Table S1 Additional file 3: Table S2 and Additional file 4: Table S3. Upon request, a complete dataset in the form of a MaxQDA file including the included papers and the full coding file, can be shared.
