# Supplementary material for: Delineating family needs in the transition from hospital to home for children with medical complexity: part 1, a meta-aggregation of qualitative studies
Source: Orphanet J Rare Dis. 2023 Dec 12;18:386. doi: 10.1186/s13023-023-02942-9 (PMC10714518; doi:10.1186/s13023-023-02942-9)
Supplement: Supplementary file 1 — Additional file 1: PIC(o), inclusion criteria and search strategy. [file 13023_2023_2942_MOESM1_ESM.pdf]

Additional file 1. *PIC(o), inclusion criteria and search strategy*

**PIC(o)**	
**P**opulation/types of participants	Parents of children aged 0-18 years with medical complexity (CMC) as defined by Cohen et al as children with one or more complex chronic illness that requires specialized care; functional disabilities; high healthcare utilization; and high family-identified needs(1).
Phenomena of **I**nterest	The needs, wishes and experiences of parents of children with medical complexity during the transition[#] of care from a hospital setting towards home.
**C**ontext	This review will consider any study that has been performed within a healthcare system that provides both hospital care and complex home care.
**Review question**	What are the needs, preferences and experiences of parents of children with medical complexity during transition from the hospital towards home?

[#]Transition is described as a passage from one life phase, condition, or status to another. A multiple concept embracing the elements of process, time span, and perception. The characteristics of transition include: (a) the movement from one phase to another (b) the disruption due to the loss of an expectation (c) the perception because of individualized meaning, and (d) the response to the transition(2).

**Inclusion criteria**	**Exclusion criteria**
Parents of children with medical complexity(1) aged 0-18 years.	Studies focussing on hospital to home transition of parents of healthy children.
	Studies focussing on transition of care from paediatric care towards adult care.
	Studies focussing solely on adult healthcare.
	Studies focussing on transition from hospital towards nursing homes and settings other than the home.
	Studies focussing on needs, wishes and experiences of professionals during hospital to home transition.

1. Cohen E, Kuo DZ, Agrawal R, Berry JG, Bhagat SK, Simon TD, et al. Children with medical complexity: an emerging population for clinical and research initiatives. Pediatrics. 2011;127(3):529-38.
2. Chick N, Meleis AI. Transitions: A nursing concern. In: Chinn PL, editor. Nursing Research Methodology. Boulder, CO: Aspen Publication; 1986. p. 237-57.

Search strategy

MEDLINE (Ovid):

#	Searches
1	Transitional Care/ or Patient Transfer/ or Patient Discharge/ or (exp Child Health Services/ and Tertiary Care Centers/) or (transition* or discharge or co-ordinated service* or coordinated service*).ti,ab,kw.
2	(transfer* adj2 (care* or patient)).ti,ab,kw.
3	1 or 2
4	exp Home Care Services/ or Hospice Care/
5	(medical home* or home care or unit* or clinic or service* or program* or framework* or model or hospice).ti,ab,kw. or home.ti.
6	((hospital or transition* or discharge or nicu) adj3 home).ti,ab,kw.
7	4 or 5 or 6
8	Needs Assessment/ or "Health Services Needs and Demand"/ or Educational Status/ or exp Self Care/ or self efficacy/ or (education or psychology).fs.
9	(need* or challeng* or prefer* or wish* or obstacle* or worry or worries or confidence or experienc* or satisf* or empower* or self-management or education or perception*).ti,ab,kw.
10	8 or 9
11	exp Parents/ or Parenting/ or Caregivers/ or Family/
12	(parent* or caregiver* or family or families or familial or mother* or father*).ti,ab,kw.
13	11 or 12
14	Disabled Children/
15	(exp Intensive Care Units, Pediatric/ or exp Emergency Service, Hospital/) and Patient Discharge/
16	(chronic* or medical complex* or medically complex* or comorbid* or co-morbid* or multiple morbidit* or complex medical need* or complex medical condition* or special medical condition* or complex health condition* or complex need* or complex care* or complex care need* or complex health need* or complex healthcare need* or complex health care need* or CSHCN or CSHCNs or CMC or yshcn* or disabled child* or medical fragil* or resource intensive need* or extensive health care need* or technology dependen* or complex technological health care need* or complex

	technological healthcare need* or special health care need* or complex multidisciplinary health*).ti,ab,kw.
17	((medical* or need* or health condition* or care or disease*) adj3 complex*).ti,ab,kw.
18	((special or extensive) adj3 need*).ti,ab,kw.
19	14 or 15 or 16 or 17 or 18
20	exp Pediatrics/ or exp Child/ or exp Infant/
21	(child* or infan* or neonat* or newborn* or baby or babies or preschool or preteen* or schoolchild* or pediatric* or paediatric* or juvenile or toddler* or kids or girl* or boy* or school age* or minors).ti,ab,kw.
22	20 or 21
23	3 and 7 and 13 and 19 and 22
24	limit 23 to "qualitative (maximizes sensitivity)"
25	qualitative research/ or empirical research/ or grounded theory/ or interview/ or Interviews as Topic/ or Narration/ or focus groups/
26	(qualitative or experience* or interview* or ethnograph* or action research or ethnological or grounded theor* or grounded research or grounded stud* or hermeneutic* or naturalistic inquir* or narrative analysis or participant observ* or phenomenol* or life stor* or focus group* or fieldwork or field work or key informant or structured clinical program or chronologic plan or survey* or empiric*).ti,ab,kw.
27	((semi-structured or semistructured or unstructured or informal or in-depth or indepth or face-to-face or structured or guide) adj2 (interview* or discussion* or questionnaire*)).ti,ab,kw.
28	(glaser adj2 strauss).ti,ab,kw.
29	25 or 26 or 27 or 28
30	23 and 29
31	24 or 30
32	Transition to Adult Care/ or ((adult healthcare or adult health care or adult care or adult service* or young adult* or adulthood) not parent*).ti,ab,kw. or (transition to adult care or transition from child to adult health service* or transition from pediatric to adult care).ti,ab,kw.
33	31 not 32

PSYCINFO (OVID):

#	Searches
1	health care services/ or hospital discharge/ or discharge planning/ or (transition* or discharge or co-ordinated service* or coordinated service*).ti,ab,id.
2	(transfer* adj2 (care* or patient)).ti,ab,id.
3	1 or 2
4	home care/ or hospice/
5	(medical home* or home care or unit* or clinic or service* or program* or framework* or model or hospice).ti,ab,id. or home.ti.
6	((hospital or transition* or discharge or nicu) adj3 home).ti,ab,id.
7	4 or 5 or 6
8	parents/ or fathers/ or mothers/ or parenting/ or caregivers/ or family/
9	(parent* or caregiver* or family or families or familial or mother* or father*).ti,ab,id.
10	8 or 9
11	exp multiple disabilities/ or disabilities/ or disability management/ or neonatal intensive care/ or (chronic* or medical complex* or medically complex* or comorbid* or co-morbid* or multiple morbidit* or complex medical need* or complex medical condition* or special medical condition* or complex health condition* or complex need* or complex care* or complex care need* or complex health need* or complex healthcare need* or complex health care need* or CSHCN or CSHCNs or CMC or yshcn* or disabled child* or medical fragil* or resource intensive need* or extensive health care need* or technology dependen* or complex technological health care need* or complex technological healthcare need* or special health care need* or complex multidisciplinary health*).ti,ab,id.
12	pediatrics/ or child care/ or chronically ill children/ or (child* or infan* or neonat* or newborn* or baby or babies or preschool or preteen* or schoolchild* or pediatric* or paediatric* or juvenile or toddler* or kids or girl* or boy* or school age* or minors).ti,ab,id.
13	3 and 7 and 10 and 11 and 12
14	limit 13 to "qualitative (maximizes sensitivity)"
15	exp qualitative methods/ or exp empirical methods/ or interviews/ or exp focus group/ or semi-structured interview/ or interpretative phenomenological analysis/ or exp interviewing/ or narrative analysis/ or participant observation/ or questioning/ or

	thematic analysis/ or exp Narratives/ or exp Storytelling/ or exp "Experiences (Events)"/
16	(qualitative or experience* or interview* or ethnograph* or action research or ethnological or grounded theor* or grounded research or grounded stud* or hermeneutic* or naturalistic inquir* or narrative analysis or participant observ* or phenomenol* or life stor* or focus group* or fieldwork or field work or key informant or structured clinical program or chronologic plan or survey* or empiric*).ti,ab,id.
17	((semi-structured or semistructured or unstructured or informal or in-depth or indepth or face-to-face or structured or guide) adj2 (interview* or discussion* or questionnaire*)).ti,ab,id.
18	(glaser adj2 strauss).ti,ab,id.
19	15 or 16 or 17 or 18
20	3 and 7 and 10 and 11 and 12 and 19
21	14 or 20
22	(adult care or adult* or midwifery or child abuse).ti,ab,id. or child abuse/ or public health/ or exp military personnel/ or exp "racial and ethnic groups"/ or exp criminal behavior/
23	21 not 22

CINAHL (Ebsco):

((MH "Transitional Care") OR (MH "Discharge Planning+") OR (MH "Patient Discharge Education")) OR (MH "Child Health Services+/AM/OG") OR TI (transition* or discharge or co-ordinated service* or coordinated service*) OR AB (transition* or discharge or co-ordinated service* or coordinated service*)

AND

((MH "Home Health Care") OR (MH "Hospice Care") OR TI home*) OR (TI (medical home* or home care or unit* or clinic or service* or program* or framework* or model or hospice) OR AB (medical home* or home care or unit* or clinic or service* or program* or framework* or model or hospice)) OR (TI ((hospital or transition* or discharge or nicu) N3 home) OR AB ((hospital or transition* or discharge or nicu) N3 home))

AND

((MH "Needs Assessment") OR ((MH "Health Services Needs and Demand+")) OR (MH "Parental Role+") OR (MH "Self Care+") OR (TI (need* or challeng* or prefer* or wish* or obstacle* or worry or worries or confidence or experienc* or satisf* or empower* or self-management or education or perception*) OR AB (need* or challeng* or prefer* or wish* or obstacle* or worry or worries or confidence or experienc* or satisf* or empower* or self-management or education or perception*))

AND

(((MH "Parents") OR (MH "Fathers") OR (MH "Mothers")) OR (MH "Caregivers") OR (MH "Family") OR (MH "Parents of Disabled Children")) OR TI (parent* or caregiver* or family or families or familial or mother* or father*) OR AB (parent* or caregiver* or family or families or familial or mother* or father*)

AND

((MH "Child, Disabled") OR (((MH "Intensive Care Units, Pediatric+") OR (MH "Emergency Service+")) AND (MH "Patient Discharge+")) OR (TI (chronic* or medical complex* or medically complex* or comorbid* or co-morbid* or multiple morbidit* or complex medical need* or complex medical condition* or special medical condition* or complex health condition* or complex need* or complex care* or complex care need* or complex health need* or complex healthcare need* or complex health care need* or CSHCN or CSHCNs or CMC or yshcn* or disabled child* or medical fragil* or resource intensive need* or extensive health care need* or technology dependen* or complex technological health care need* or complex technological healthcare need* or special health care need* or complex multidisciplinary health*) OR AB (chronic* or medical complex* or medically complex* or comorbid* or co-morbid* or multiple morbidit* or complex medical need* or complex medical condition* or special medical condition* or complex health condition* or complex need* or complex care* or complex care need* or complex health need* or complex healthcare need* or complex health care need* or CSHCN or CSHCNs or CMC or yshcn* or disabled child* or medical fragil* or resource intensive need* or extensive health care need*

or technology dependen* or complex technological health care need* or complex technological healthcare need* or special health care need* or complex multidisciplinary health*))) OR (TI ((medical* or need* or health condition* or care or disease*) N3 complex*) OR AB ((medical* or need* or health condition* or care or disease*) N3 complex*)) OR (TI ((special or extensive) N3 need*) OR AB ((special or extensive) N3 need*))

AND

(MH "Pediatrics+") OR (MH "Child+") OR (MH "Infant+") OR TI (child* OR infan* OR neonat* OR newborn* OR baby OR babies OR preschool OR preteen* OR schoolchild* OR pediatric* OR paediatric* OR juvenile OR toddler* OR kids OR girl* OR boy* OR school age* OR minors) OR AB (child* OR infan* OR neonat* OR newborn* OR baby OR babies OR preschool OR preteen* OR schoolchild* OR pediatric* OR paediatric* OR juvenile OR toddler* OR kids OR girl* OR boy* OR school age* OR minors)

AND

((MH "Qualitative Studies+") OR (MH "Empirical Research")) OR ((MH "Interviews+") OR (MH "Focus Groups") OR (MH "Narratives+")) OR (TI (qualitative or experience* or interview* or ethnograph* or action research or ethnological or grounded theor* or grounded research or grounded stud* or hermeneutic* or naturalistic inquir* or narrative analysis or participant observ* or phenomenol* or life stor* or focus group* or fieldwork or field work or key informant or structured clinical program or chronologic plan or survey* or empiric*) OR AB (qualitative or experience* or interview* or ethnograph* or action research or ethnological or grounded theor* or grounded research or grounded stud* or hermeneutic* or naturalistic inquir* or narrative analysis or participant observ* or phenomenol* or life stor* or focus group* or fieldwork or field work or key informant or structured clinical program or chronologic plan or survey* or empiric*)) OR (TI ((semi-structured or semistructured or unstructured or informal or in-depth or indepth or face-to-face or structured or guide) N2 (interview* or discussion* or questionnaire*)) OR AB ((semi-structured or semistructured or unstructured or informal or in-depth or indepth or face-to-face or structured or guide) N2 (interview* or discussion* or questionnaire*))) OR (TI glaser N2 strauss OR AB glaser N2 strauss)

NOT

((TI midwifery OR AB midwifery) OR (TI adult* OR AB adult*))
