# Supplementary material for: Delineating family needs in the transition from hospital to home for children with medical complexity: part 1, a meta-aggregation of qualitative studies
Source: Orphanet J Rare Dis. 2023 Dec 12;18:386. doi: 10.1186/s13023-023-02942-9 (PMC10714518; doi:10.1186/s13023-023-02942-9)
Supplement: Supplementary file 2 — Additional file 2: Table S1. Included study characteristics. [file 13023_2023_2942_MOESM2_ESM.pdf]

Table 1. *List of included studies and demographics of infants and parents*

		Infant demographic							Parents demographic				
First author	Year	Infants	Female	Age at discharge	Medical condition[X]	Medical technology[X]	Time in hospital prior to discharge	Moment(s) of data collection	Inteviewed Parents	Interviewed Mothers	Age parents	Education (High school or less)	Other specifics
		n	n (%)	Range in mo (n), Md/Mn, SD (if available)	Diagnosis (n)	Device (n)	Range (n), MD/Mn, SD (if availaboe)	weeks (unless specified otherwise) after discharge	n	n (%)	Range in y (n), Md/Mn, SD (if available)	n (%)	
Aasen	2018	9	N/A	0-17 (9)	Ca (2), synd (2), Various infections (5)	N/A	N/A	N/A	9	6 (67)	39	N/A	Hospital at home (HAH); hospital services at home
Barone	2020	8[A]	N/A	0-6 (8)	PREM and/or complications related to PREM (5), specific syndrome (3)	CVL (1); g-tube (6); LFNC (3), CR monitor (n=1), Subgaleal shunt (1), O2 (4), Glucometer (1)	NICU for >28d or PICU for >14d (8)	1mo	N/A	N/A	<25 (1), 25-35 (6)	1 (14)	
Berman	2018	18[B]	5 (28)	N/A	N/A	NG-tube (10), POX (2), O2 (1) or Sa (1) monitor, GJ- tube (1), G-tube (3), feeding pump (1), suction device (2), Kangaroo joey (1), O2 (4), Trach (2), Vent (1), NEB (1), VP shunt (1)	N/A	N/A[H]	15	14 (93)	22-29 (6), 30-40 (6), 40-47 (3) (1 = N/A)	2 (13)	NICU hospitalization
Brenner	2015	15	7 (47)	1–11 (7), Mn 5.7, 2.96 SD	N/A	Trach (15), Ventilator (2), CPAP (13)	3-22mo (15), Mn 8.7mo, 6.36 SD	<5 y	15	12 (80)	N/A	N/A	Most children (n=13) were discharge to home with a homecare package
Callans	2016	N/A	N/A	N/A	Compromised airway	Trach (N/A)	N/A	N/A[I]	18	16 (89)	N/A	N/A	
Coelho Ramos	2015	N/A	N/A	35-108 (N/A)	congenital causes (8), acquired causes (2)	N/A	N/A	N/A	10	10 (100)	N/A	N/A	
Cohen	1999	5[C]	N/A	<204 (5)	N/A	Ventilator support (5)	17 mo (1)	48-72h, 3mo, 7mo, 12mo[J]	N/A	N/A	N/A	N/A	Families from socially marginalized groups (n=3)
Desai	2015	18	7 (39)	0-23 (2), 24-48 (6), 60-144 (6), 156-216 (4)	No chronic conditions (6), Non-complex chronic conditions (3), Medical complexity (9)[G]	G-tube (1 or more)	< 3d (16), > 3d (2)	< 3mo	18	17 (94)	18-24 (2), 25-34 (4), 35-44 (7), >45 (5)	4 (22)	
Fairless	2021	8[B]	N/A	43w GA (38-48w)	Born prematurely <34w GA, discharged with >1 medical condition, >1 medication and medical equipment at home (not specified per child)	N/A	N/A	< 3y	6	6 (100)	>18y	N/A	
Gaskin	2018	12	7 (58)	0-1 (7)	HLHS (10), HRHS (1), TOF (1), Noncardiac defects (5), Genetic abnormaility (3)	Mechanical ventilation (6) (pre-operative management)	N/A	2, 8[K]	16	12 (75)	20-25 (3), 26-30 (4), 31-40 (5)	4 (33)	Children underwent stage 1 treatment for complex CHD
Gaskin	2021	12	7 (58)	0-1 (7)	HLHS (10), HRHS (1), TOF (1), Non-cardiac defects (5), Genetic abnormaility (3)	N/A	N/A	2, 8[K]	16	12 (75)	20-25 (3), 26-30 (4), 31-40 (5)	4 (33)	Children underwent stage 1 treatment for complex CHD
Hebballi	2021	16	N/A	N/A	Former NICU patients with an extended NICU stay, having complex medical conditions	N/A	N/A	Once after discharge, not specified when	16	15 (94)	N/A	N/A	Combination of focus groups and individual interviews
Lakshmanan	2019	21	N/A	6-12 (21)	Imp. anus (1), Torticolis (3), CP (1), DD (11), CM of Optic nerve (1), EP (1), HP (1), MEG (1), Disorder of psychological development (1), CVI (1), ET (1), Optic atrophy (1), BPD (4), Anoxic brain damage (1), Language DD (3), Plagiocephaly (1), EA (2), VACTERL (2), Cleft palate/lip (1), Multiple congenital anomalies (1), OSA (2), Other congenital malformations of abdominal wall (1), Other reduction abnormalities of brain (1), RD (1), VUR (1), PH (1), IPD (1), SNHL (1), MD (1), Absence of rectum (1), HT (1), Mediastinitis (1), COA (1), RA (1)	Medical equipment (10), O2 (5), G-tube (7), Trach or ventilator dependence (1), VP-shunt (1)	31-135d (21), Md 83d	N/A	21	20 (95)	Md 26, IQR 23-27	N/A	LIving in a neighborhood with 3-4th quartile economic hardship (n=13)
Leary	2020	20	12 (60)	0-180 (20)	Neur (7), CV (3), GI (3), HEME (3), Malignancy (2), RES (1), Metabolic (1) complex chronic condition	N/A	N/A	5d	N/A	N/A	N/A	N/A	Data collected at readmission
Lerret	2017	48	24 (50)	0-210 (48), Md 33.6	Liver (20), heart (15), kidney (8), multivisceral (5), lung (1) transplant	Enteral/tube feeding (17), CVL (14), Drainage tube (3)	4-90d (48), Mn 13.5d	3w, 3mo, 6mo	48	41 (85)	19-55 (48), Md 34	N/A	
Leyenaar	2017	23	10 (44)	1-14 (23), Md 5.5	CA or noncancer multisystem disease (23)	g-tube, j-tube, CVL, trach, ventilator, VP-shunt, ostomies, or dialysis (15)	N/A	N/A[L]	23	19 (83)	33-45 (23), Md 38	3 (14)	
Manhas	2012	2	2 (100)	<60 (2)	N/A	Ventilator support (2)	N/A	N/A	2	2 (100)	N/A	N/A	Receiving provincially funded home-care services
Margolan	2004	15	N/A	36-228 (15)	CH (1), muscular dystrophy with hypomylenation (1), Congenital myopathy (1), SMA (3), PRS (1), Lung disease (1), Severe sco (1), TM (1), BM (1), Midline defects (1), Congenital hypoventilation syndrome (2), Acquired hypoventilation (11), DAA resulting in TM (1), DH (1), Spinal injury (1), CMG (1), EP (1)	Ventilator support >3mo (15), NEB (2), G-tube (6), NG-tube (1)	Mn 513d	N/A	16[O]	13 (81)	N/A	N/A	Home care support (n=13)
Murdoch	2011	10[D]	4 (40)	0[F]	PREM (8), CLD (4), PDA (4), RDS (2), Hirschprung's (2), Meconium plug syndrome (1), Jaundice (2), Trisomy 21 (1), GORD (2)	O2 (3), Colostomy care (2), Daily bowel washouts (1)	6-238d (10), Md 49d	3-15mo, Md 8mo	9	9 (100)	27-42 (9), Md 34	N/A	NICU hospitalization
Norton	2021	8	3 (37.5%)	0-2y	PREM (8), not specified per child	Nasogastric feeding tube (25%), gastric feeding tube (37.5%), supplemental oxygen via nasal tube (62.5%)	112d (60-160)	<2y	8	8 (100)	35 (26-42)	2 (25)	
Okido	2012	1	0	48 (1)	Unspecified encephalopathy of unclear cause (1)	G- tube (1), Metallic trach (1)	6 months (1)	+- 3y	1	1 (100)	37 (1)	1 (100)	Rehospitalized several times due to pneumonia 2y later
Okido	2015	12	N/A	<144 (12)	N/A	G-tube or GJ-tube (11), Trach, non-invasive mechanical ventilation, or O2 therapy (7), Urinary relief catheter, or intestnal washing (3)	N/A	N/A	12	12 (100)	Mn 34	12 (100)	
Silva-Rodrigues	2019	11	N/A	24-204 (11)	ALL (11)	N/A	N/A	N/A[M]	11	9 (82)	29-48 (11), Md 37	11 (100)	Children in the induction period of oncological treatment. Parents already provided home care to their child
Simeone	2018	N/A	N/A	N/A	CHD (24)	Device the Deans (1)	3-120d, Mn 37d, SD 25.39	1-3mo[N]	24	18 (75)	18-49 (24), Mn 37.18 SD 8.976	12 (50)	
Zanello	2015	17[E]	9 (53)	3-68 (17), Md 9.1	PREM <1000 gr (9), Encephalopathy (5), Hydrocephalus (1), Myopathy (1), CM (1)	N/A	N/A	0.7-8.3w, Mn 3.8 SD 2.4; Md 3.5	23	15 (65)	N/A	N/A	

y: Years; mo: Months; w: Weeks; d: Days; Md: Median; Mn: Mean; SD: Standard Deviation; ALL: Acute lymphoblastic leukemia; BM: Bronchomalacia; BPD: Bronchopulmonary dysplasia; Ca: Cancer; CH: Cerebellar hypoplasia; CHD: Congenital heart disease; CHS: Congenital hypoventilation syndrome; CLD: Chronic lung disease; CM: Congenital Malformations; CMG: Congenital myaesthenia gravis; COA: Coarctation of Aorta; CP: Cerebral palsy; CPAP: Continuous Positive Airway Presure; CR monitor: Cardiorespiratory monitor; CV: Cardiovascular; CVI: Cortical visual impairment; CVL: Central venous line; DAA: Double aortic arch; DD: Developmetatal delay; DH: Diaphragmatic hernia; EA: Esophageal Atresia; EP: Epilepsy; ET: Esotropia; G-tube; Gastrostomy tube; GI: Gastrointestinal; GJ-tube: Gastro-jejunostomy tube; GORD: Gastro-oesophageal reflux disease; HEME: Hematologic; HLHS: Hypoplastic left heart syndrome; HP: Hemiplegia; HRHS: Hypoplastic right heart syndrome; HT: Hypertension; Imp. Anus: Imperforate Anus; IPD: Interstitial pulmonary disease; LFNC: Low-flow nasal cannula; MD: Motor delay; MEG: Megalencephaly; MET: Metabolic; NEB: Nebulizer; Neur: Neurological; NG-tube: Nasalgastric tube; NICU: Neonatal intensive care unit; O2: Oxygen; OSA: Obstructive sleep apnea; PDA: Patent ductus arteriosus; PICU: Pediatric intensive care unit; PH: Pulmonary hypoplasia; POX: Pulse oximeter; PREM: Prematurity; PRS: Pierre Robin syndrome; RA: Renal agenesis; RD: Renal dysplasia; RDS: Respiratory distress syndrome; RES: Respiratory; Sa: Saturation; SMA: Spinal muscular atrophy; SNHL: Sensorineural hearing loss; synd: Syndrome; TM: Tracheomalacia; TOF: Tetralogy of Fallot; Trach: Tracheostomy; VP shunt; Ventriculoperitoneal shunt; VUR: Vesicoureteral reflux; A: Including twins; B: including 2xtwins, 1xtriplets; C: (including one child that dropped out because they deceased; D: including twins; E: 16 families; F: Median gestational age 31 weeks (range 24-42); G: Pediatric medical complexity algorithm (PMCA) category; H: Average age at data collection was 34 months; I: Inserted tracheostomy tube over the past 2 years; J: And before discharge; K: And before and after stage 2 surgery; L: During hospitalization (in pilotphase 1 to 2 weeks after hospitalization); M: time of therapie varied from 21 to 75 days; N: just returned home from hospital after undergoing cardiac surgery; O: Including 1 foster parent; X: Could be more than 1 per individual
