# Supplementary material for: Delineating family needs in the transition from hospital to home for children with medical complexity: part 1, a meta-aggregation of qualitative studies
Source: Orphanet J Rare Dis. 2023 Dec 12;18:386. doi: 10.1186/s13023-023-02942-9 (PMC10714518; doi:10.1186/s13023-023-02942-9)
Supplement: Supplementary file 3 — Additional file 3: Table S2. Overview of the synthesized findings, categories and a selection of study findings. Illustrated by citations. [file 13023_2023_2942_MOESM3_ESM.pdf]

Additional file 3: Table 2. *Overview of the synthesized findings, categories, and a selection of study findings. Illustrated by citations.*

Synthesized findings (n=9)	Categories (n=50)<br>Number and level of credibility of findings per category*	Findings (n=402)<br>A selection of findings per category	Citations
**1 Parental empowerment: shifting from care recipient to caregiver**	Development of own practices (10F, U+C)	Growing competence in caregiving tasks<br><br>Becoming an expert caregiver by experience<br><br>Encourage parents to learn as soon as possible	"I am now going to be responsible for keeping my child alive on an hourly basis. We're in charge here and we are responsible and we have to figure this out."<br><br>"Don't let mum and dad cower in the corner."<br><br>"I know my child best. I want to drive the bus."<br><br>"Before we came home fully they gave us stepping stones. We brought him out for a day and came back and that happened twice. Leave the hospital, come back for the night and maybe the next day we went out again. And eventually we left."
	Emotional process (14F, U+C)	Despairing learning process<br><br>Coping with challenges improves own future orientation<br><br>Vigilance becomes less evident over time	
	Growing responsibility (13F, U+C)	Balance between confidence and knowing when to seek help<br><br>Becoming an advocate for peers/professionals	
**2 Coordination of care**	Coordination needs during transition from hospital to home (6F, U+C)	Make parents feel supported in the transition<br><br>Parents actively involved in the planning of home care	"I think that it is important to make parents feel that they are supported in the transition. We had the first meeting with all those professionals and we thought: 'That's great! Finally, they will really accompany us all the way'. Then, that's not true."<br><br>"We received information about the plan and the nurses showed us how to do things. They also informed the community nurse, so yes, they prepared everything well and then we felt more ready to go."<br><br>"The 'post' (discharge) is a no man's land."
	Coordinating follow up (15F, U+C)	Clear contact after discharge is helpful<br><br>Involve caregivers in the transfer process	
	Incorporating (digital) coordination tools into care plan (4F, U+C)	Mobile health technology could help discharge planning	
**3 Communication and information**	Comprehensible (11F, U+C)	Explaining issues in a comprehensible manner and language	"The skilled people do not realize that, as unskilled person, you need to know the reason why you have to do something, because in that way, if nothing else, you internalize it."
	Reliable and available (5F, U+C)	Reliable information should be available	

		There should be ample opportunity to ask	
	Consistent (10F, U+C)	Discontinuity of care leads to poor communication	"In our NICU, there was a new doc that came through almost daily, so I didn't feel I had anyone on the medical side that was giving me clear and consistent information. I didn't feel there was a unified plan from the team."<br><br>"When we left, the plan was for him to eat orally during the day and be fed by night (G-tube). He's never ate orally since the surgery so that was completely and totally unexpected."<br><br>"I think video chat would be awesome…can you see what I see for example with g-tubes?"
	Customized (2F, C)	Customized information makes parents feel safe and in control	
	Clear expectations (6F, U+C)	Consistency of expectations at time of discharge<br><br>Set appropriate expectations about inevitable setbacks	
	Use of information tools (3F, U)	Gaining knowledge from external sources (i.e. books, internet)<br><br>Health portals to communicate and educate	
**4 Training skills**	Gradual education (7F, U+C)	Gradual education results in safe and competent care<br><br>Empirical learning (trial-and-error)	"It was a HUGE learning curve figuring out once, so okay now that you have identified that your baby's sick, what does that mean? [ ] We began to get to know our baby, interpret different signals, but it took quite a while to figure it out."<br><br>"Sometimes I forgot to give my child the medicine." [ ] The phenobarbital has a very bad taste and he does not like it, it's very difficult"<br><br>"…[ ] I have to sort of try, and by a process of elimination work out which [cry] it is, and try to sort it out, or he may be general tired and over stimulated, he seems to get over stimulated quite easily."
	Practice (4F, U+C)	Plan protected practice time without child<br><br>Opportunity to practice home care tasks timely	
	Personalized training (3F, U+C)	Identify knowledge and skill gaps and develop capability<br><br>Create adapted, tailor-made programs	
	Anticipation of post discharge situation (10F, U+C)	Prepare for the unexpected<br><br>Plan in case of deterioration at home Administering medicine creates difficulties	
	Tackle practical issues (3F, U+C)	Gain adequate knowledge of potential equipment issues	
**5 Preparation for discharge**	Time (4F, U+C)	Set time for discharge moment (early in day)	"I know there's never really a set time. But we were told that we'd probably be out of here by noon at the latest. We didn't leave until like 7:30 that night."
	Equipment (6F, U+C)	Set up equipment in the home before discharge<br><br>Equipment may be different at home (confusing)	

	Accommodation (3F, C)	Substandard housing and housing adjustments delay discharge	“Before you get home you have to have everything set up and you need to figure out where everything goes because it was such a scary experience. You’re so confused with just getting home.”<br><br>“We were ‘out of sight, out of mind’. Coming home to medical bills added to the other stressors associated with the transition home”.
	Transportation (2F, U+C)	Check transportation arrangements	
	Finances (2F, U+C)	Timely inventory of financial situation before discharge	
6 Access to resources and support system	Home professionals (7F, U+C)	Hard to find good paid caregivers<br><br>Arranging homecare takes time	“A few of them [nurses] left, because they found a full-time job and couldn’t get full-time with homecare. Another quit because she couldn’t handle then nights. I trained another one, she did the training but never came back. I can’t say was surprised.”<br><br>“We maybe spent a bit more on petrol and things like that, because of travelling to hospital and stuff.”<br><br>“Sometimes, if the insurance is covering, that’s it. But sometimes, you need the doctor to write the prescription and send it to the insurance. If I need them right away, I buy out of pocket.”<br><br>“It is hard to find families like us.”<br><br>“It is such an important part of transition to connect parents in the NICU to parents outside the NICU who have been through things. Apart from all the medical challenges I am facing, it is so hard to find that part of the community.”<br><br>“We didn’t communicate very much, I guess because we were both very tired.”<br><br>“We’re starting to create a routine that will work for both of us. I think it’s helped our relationship so we’re not arguing so much now.”
	Distance to healthcare (4F, U+C)	Distance to a (good) hospital in case of emergency	
	Access to services (7F, U+C)	Dependency on a specific hospital due to complexity<br><br>Availability of complex medication at ‘normal’ pharmacy	
	Financial support programs (12F, U+C)	Struggle to retain funding after discharge<br><br>Financial issues force families to prioritize	
	Friends and family support system (16F, U+C)	Attention to siblings by grandparents<br><br>Hard to find committed second caregiver/care difficult for relatives due to complexity	
	Peer support system (12F, U+C)	Peer support groups help connect with other parents with similar experience and receive helpful advice<br><br>Peer support to help accept new reality	
	Official institutions support system (2F, C)	Reliant on formal services to obtain a break in caring	
	Emotional/psychological support (6F, U+C)	Make parents aware of emotional support and encourage them to use it	
	Educational provision (2F, C)	Disputes about funding of caretakers at school	
	Religion and alternative therapies (2F, U+C)	Complementary and alternative therapies can be helpful to cope	

**7 Emotional experiences: fatigue, fear, isolation, and guilt**	Fatigue (8F, U+C)	Chronic psychophysical fatigue	"Now, as it is now a bit better… but I've always been tired.. my body, my mind."<br><br>"You think that here (hospital) you are safe. So at first, I was scared, a little apprehensive, I was afraid of having to go back at any time, of something happening and not realizing it."<br><br>"If there was more of a prep that you have been through a traumatic process, you have looked into the abyss of whether your child will live – that is not something most parents go through. Be gentle with yourself."<br><br>"I think I sat for 24 hours with him downstairs in intensive care and… the images… won't leave my head… and that's what's like torture."<br><br>"This is not the life I had planned."<br><br>"I genuinely don't know about the future, it is something that we don't let ourselves think about because it would make us more depressed. It is a very, very difficult situation to be in."
	Fear, anxiety and being overwhelmed (34F, U+C)	Overwhelmed and petrified first days at home<br><br>Fear of child's death<br><br>Fear of the unknown	
	Feelings of isolation (11F, U+C)	Social isolation because network cannot relate<br><br>Lack of social interaction is depressing	
	Anger, guilt and grief (9F, U+C)	Angry about disruption of planned life (and feeling guilty about feeling angry)<br><br>Bereavement left grief, confusion and trepidation<br><br>The search for the causes and people to blame	
	Future perspective (8F, U+C)	Difficulty thinking beyond the present moment<br><br>Unknown future is frightening<br><br>Fear of stigmatization	
**8 Parent-professional relationship**	Equality between parent and professional (8F, U+C)	Share responsibility with staff at home<br><br>Shifting boundaries between personal and professional relation	"Everyone telling you how to do things is very disempowering."<br><br>"Whenever I have any doubt, they (nurses) provide good guidelines, all things related to care, medication, the effect of medication."<br><br>"Our pediatrician used to be one of her doctors at the NICU. So that was an advantage, she knew what she needed, she didn't need to ask questions, she knew what's good for her."
	Special bond between professional and child (4F, U)	Nurses see the child's individual needs	
	Strength of relational continuity (7F, U+C)	Familiar caregiver for follow up<br><br>Complexity of disease makes recognition of symptoms difficult for new professionals	
	Parental acknowledgement (17F, U+C)	Involvement of parent in decision making<br><br>Parents feel stressed about repeated 'discharge test'	

	Concern about level of expertise of the professional (5F, U+C)	Providers who are knowledgeable about the care plan	"I really feel like I need to be on constant watch because a lot of mistakes have been made. I've gotten to the point that whenever anyone walks into her room, I immediately ask 'who are you, what are you here to do and who told you to do it'."
	Healthcare professional's attitudes (7F, U+C)	Parental-professional role assumed by professionals	
**9 Changing perspective: finding (new) routines and practices**	Organize home environment (9F, U+C)	Adjusting to all the equipment at home<br><br>Maintain home environment despite equipment	"It took us so long to get comfortable with our setup at home. It's the different machines, we rearranged his whole room."<br><br>"Our life turns upside down, because they become the center of our lives."<br><br>"We were very much taken care of in the hospital; however, we got a completely different sense of calm at home, and we often slept and relaxed better."<br><br>"All of us leaving as a family…yes that was nice. Something she [sibling] shared as well."<br><br>"This underlying state of constant stress has definitely honed my ability to think quickly in all other areas of my life. I have absolutely no trouble making decisions about anything these days."<br><br>"After everything we've been through, probably the hardest thing for us is letting her go be a kid. We'd like to keep her in our safe little bubble, but she's doing so well. It is time to let her be a normal kid."
	Lack of privacy and control (4F, C)	Stressful to have caregivers in the home, lack of privacy	
	Establishing new routines (7F, U+C)	Establish new routines to organize child's care<br><br>Finding (new) routines at home reduced stress	
	Disruptions of routines (6F, U+C)	Admission changes both parent and child<br><br>Impact of (new) hospitalization on family routine	
	Parents' dual role and the importance of self-care (13F, U+C)	Balance care for sick child and self-care<br><br>Competing responsibilities with siblings/household/job	
	Adjusting to having a special need child (14F, U+C)	Finding a new normal by comparing your social context<br><br>Comments from strangers about child<br><br>Fear of vulnerability complicates return to normal activities	
	Resume normal family life at home (18F, U+C)	Whole family focuses on the needs of the sick child<br><br>Enjoy non-medical moments in daily/family life<br><br>Be together as a family again at home	

*A level of credibility that was applied to all study findings: F=findings; U=Unequivocal; C=Credible
