# Supplementary material for: Delineating family needs in the transition from hospital to home for children with medical complexity: part 1, a meta-aggregation of qualitative studies
Source: Orphanet J Rare Dis. 2023 Dec 12;18:386. doi: 10.1186/s13023-023-02942-9 (PMC10714518; doi:10.1186/s13023-023-02942-9)
Supplement: Supplementary file 4 — Additional file 4: Table S3. Synthesized findings represented throughout the included papers. [file 13023_2023_2942_MOESM4_ESM.pdf]

Additional file 4 – Table 3: *Synthesized findings represented throughout the included papers*

Synthesized findings (n=9) / Papers (n=25)	1 Parental empowerment: shifting from care recipient to care receiver	2 Coordination of care	3 Communication and information	4 Training skills	5 Preparation for discharge	6 Access to resources and support system	7 Emotional process: fatigue, fear, isolation, and guilt	8 Parent-professional relationship	9 Changing perspective: finding (new) routines and practices
Aasen 2018									
Barone 2020									
Berman 2018									
Brenner 2015									
Callans 2016									
Coelho Ramos 2015									
Cohen 1999									
Desai 2015									
Fairless 2021									
Gaskin 2018									
Gaskin 2021									
Hebballi 2021									
Lakshmanan 2019									
Leary 2020									
Lerret 2017									
Leyenaar 2017									
Manhas 2012									
Margolan 2004									
Murdoch 2011									
Norton 2021									
Okido 2012									
Okido 2015									
Silva-Rodrigues 2019									
Simeone 2018									
Zanello 2015									
